# Contribution of Type 2 Diabetes Susceptible Gene GCKR Polymorphisms Rs780094 and Rs1260326 to Gestational Diabetes Mellitus: A Meta-Analysis

**DOI:** 10.2174/0118715303313654241101042033

**Published:** 2025-01-09

**Authors:** Yuke Zhang, Kuangyi Wang, Chenxi Ji, Yansi Lin, Zitong Liu, Jing Chen, Feifei Zheng, Xiaoqin Yang, Yi Sun

**Affiliations:** 1 Center for Systems Biology, Department of Bioinformatics, School of Biology and Basic Medical Sciences, Suzhou Medical College of Soochow University, Suzhou 215123, China;; 2 Department of Cardiology, the Fourth Affiliated Hospital of Soochow University and Medical Center Soochow University, Suzhou 215123, China;; 3 Department of Genetics, School of Biology and Basic Medical Sciences, Suzhou Medical College of Soochow University, Suzhou 215123, China

**Keywords:** Gestational diabetes mellitus, glucokinase regulator gene, rs780094, rs1260326, meta-analysis, susceptibility

## Abstract

**Background:**

There is still no conclusive understanding of whether the glucokinase regulator (GCKR) gene rs780094 and rs1260326 polymorphisms predispose to gestational diabetes mellitus (GDM).

**Objective:**

This systematic review and meta-analysis aimed to determine the effect of the GCKR polymorphisms on GDM susceptibility.

**Methods:**

Seven literature databases were searched (from inception to February 17, 2024) to locate relevant studies included in further meta-analysis. Odds ratio (OR) and 95% confidence intervals (CI) in the pooled population were estimated to assess the effects of the variant allele on GDM risk.

**Results:**

For the rs780094 polymorphism, 13 datasets with 3443 GDM cases and 5930 nondiabetic controls were included. The pooled estimates in the allele model (OR: 1.19, 95% CI: 1.07~1.32), homozygote model (OR: 1.27, 95% CI: 1.10~1.47), dominant model (OR: 1.16, 95% CI: 1.03~1.31), and recessive model (OR: 1.31, 95% CI: 1.09~1.57) suggested that the C allele carriers were prone to GDM. For the rs1260326 polymorphism, five datasets with 1495 cases and 2678 controls were integrated. The statistically significant effect of the C allele was evident in the allele model (OR: 1.12, 95% CI: 1.01~1.24) and the homozygote model (OR: 1.26, 95% CI: 1.03~1.54).

**Conclusion:**

This meta-analysis suggested that the C allele of the rs780094 and rs1260326 polymorphisms in the GCKR gene are significantly associated with increased risk of GDM.

## 1 INTRODUCTION

Gestational diabetes mellitus (GDM) is currently the most common pregnancy complication [1] and is still increasing in prevalence worldwide [2]. This chronic hyperglycemia is a leading cause of maternal and fetal morbidity and mortality, mainly due to the development of complications, such as preeclampsia, obstetric intervention, preterm birth, and birth trauma [2]. In addition, it is associated with long-term adverse maternal and offspring outcomes, including type 2 diabetes mellitus (T2DM), cardiovascular disease, and obesity [3]. Although maternal overweight and obesity, family history of T2DM, childbearing age, physically inactive lifestyle, cigarette smoking, and ethnicity are recognized as hazardous factors for GDM [1], the exact mechanism implicated in its pathogenesis is still not fully understood. With remarkable progress in genomics, a growing body of literature suggests that genetic factors (including polymorphism) could profoundly predispose to GDM [4-6]. Therefore, tracing the genetic origin of GDM may help clarify the pathogenesis of this metabolic disorder in pregnancy.

The glucokinase regulator (GCKR) protein, encoded by the GCKR gene (location: 2p23.3), functions as a specific inhibitory regulator of the critical glucose-metabolizing enzyme glucokinase (GCK) by forming an inactive complex with it. This enzyme is a glucose phosphorylase that manipulates hepatic glucose disposal and blood glucose homeostasis [7, 8]. Therefore, genetic defects of the GCKR gene may lead to dysfunction of its protein product and contribute to dysglycemia. Several previous clinical studies suggested that plasma glucose levels, serum triglyceride concentrations, fasting insulin, and insulin resistance were associated with both the rs780094 (C > T, intronic variant) and rs1260326 (C > T, Pro446Leu) polymorphisms significantly in specific populations [9-11]. Fructose-6 (F6P) and fructose-1 phosphate (F1P) amplify or diminish GCKR-mediated inhibition, respectively. The rs1260326 variant in GCKR exhibits reduced responsiveness to physiological concentrations of F6P, indirectly leading to enhanced GCK activity [12]. The intronic locus represented by the rs780094 polymorphism is identified as an enhancer that regulates GCKR transcription in a haplotype-specific manner with other proximal variants. The alternative variant of rs780094 is likely to disrupt the transcription factor binding and consequently modulate GCKR transcriptional activity [13]. Moreover, the scattered epidemiological studies and pooled analyses declared that the GCKR rs780094 and rs1260326 polymorphisms are highly prevalent SNPs in T2DM [14-16], which shares a common genetic architecture with GDM [17]. Furthermore, our previous meta-analysis also proposed that the two common variants within the GCKR gene are associated with an elevated risk of non-alcoholic fatty liver disease (NAFLD) [18], which predisposes pregnant women to GDM [19]. These biological evidence and epidemiological investigations reinforce the hypothesis that the GCKR rs780094 and rs1260326 polymorphisms are causally linked to GDM susceptibility.

Many epidemiological studies have tried to assess the magnitude of the association between GCKR polymorphisms and GDM susceptibility, but their results are conflicting rather than consistent [20-30]. Two previous meta-analyses attempted to reach conclusive findings [31, 32]. Due to the limitations of their search strategy, one study published in Chinese [25] and two in English [22, 24] with 3196 participants were omitted in data synthesis for the rs780094 polymorphism. Furthermore, in Guo’s meta-analysis for the rs780094 polymorphism [31], three Asian datasets [23] released after the execution of this study were not integrated into its pooled results. Moreover, these two meta-analyses mistakenly categorized a Hispanic cohort from Spain into the Caucasian subgroup [26]. In the meta-analysis for the rs1260326 polymorphism [31], the pooled estimation integrated only three studies. The small sample size limited the statistical power. As some new epidemiological studies emerged, this systematic review and meta-analysis was performed to derive a more precise estimation of the association.

## 2 MATERIALS AND METHODS

### 2.1 Implementation of PRISMA Guidelines

This study rigorously followed the Preferred Reporting Items for Systematic Reviews and Meta-Analyses (PRISMA) recommendations (Table **S1**) [33]. Two independent investigative squads were formed to participate individually in every methodological procedure of this meta-analysis. All conflicts and uncertainties were resolved by consensus, and a third investigator was invited to discuss and arbitrate when necessary.

### 2.2 Literature Search

For English publications, PubMed of NCBI (https://pubmed.ncbi.nlm.nih.gov/), Elsevier Embase (www.embase.com), Web of Science of Clarivate Analytics (https://www.webofknowledge.com/), and Cochrane Library of John Wiley & Sons (https://www.cochranelibrary.com/) were queried. For Chinese literature, China National Knowledge Infrastructure (CNKI, https://www.cnki.net/), WANFANG DATA (https://www.wanfangdata.com.cn/), and China Science and Technology Journal Database (VIP, https://qikan.cqvip.com/) were searched. The retrieval formula using Boolean logic was organized by connecting the official symbol, full name, and regular alias for the GCKR gene (“Glucokinase regulator”, “Glucokinase regulatory protein”, “GCKR”, “FGQTL5”, “GKRP”), standard name and abbreviation for GDM (“gestational diabetes mellitus”, “gestational diabetes”, “GDM”), and keywords or phrases for single nucleotide polymorphisms rs780094 and rs1260326 (“SNP”, “polymorphism”, “variant”, “single nucleotide polymorphism”, “rs780094”, “rs1260326”). The literature search was finally updated on 17 February 2024.

### 2.3 Selection Criteria and Data Extraction

For an original study to be included in this meta-analysis, full-text content must be accessible as an electronic or printed copy, and the study had to meet all the following inclusion criteria: (1) human research, (2) original GDM risk study, (3) with both GDM case and control samples, (4) sufficient and accurate GCKR genotype data to assess the strength of the association. Exclusion criteria were: (1) not research article (*e.g.,* review, editorial, and abstract), (2) not human GDM study (*e.g., in vitro* or animal model research), (3) with duplicated data (in this case, only the publication with largest sample size was kept), (4) no genotype data for the GCKR rs780094 or rs1260326 polymorphism. If the genotype distributions were unavailable in the published manuscript, the corresponding authors would be contacted by email to retrieve the original data.

The following data were carefully extracted from the selected publications: name of the first author, publication date, total numbers of cases and controls, genotype data for both GDM case and control samples, country, ethnicity of the subjects, and genotyping method.

### 2.4 Quality Score Assessment

A Microsoft Excel calculator (https://accounts.smccd.edu/case/biol215/docs/HW_calculator.xls) was used to determine whether observed allelic frequency distributions of the non-GDM controls in each included dataset are consistent with the Hardy-Weinberg equilibrium (HWE). A *P*-value less than 0.05 was considered statistically significant.

The methodological quality appraisal using the Newcastle-Ottawa Quality Assessment Scale (NOS, https://www.ohri.ca/programs/clinical_epidemiology/oxford.asp, a star system with the highest score of nine stars) was conducted for each included study to comprehensively judge on three perspectives: the selection of the GDM and control groups; the comparability between the two groups; and the ascertainment of the exposure of interest (genotyping the GCKR rs780094 and rs1260326 polymorphisms). Literature with seven stars and above was considered good quality, and less than four was deemed low quality.

### 2.5 Statistical Analysis

All meta-analysis procedures were performed under an R language environment (version 4.3.2) built on the MacOS platform (version 14.2.1). The concerned statistical models and methods were implemented by the functions built in the general meta-analysis R package “meta” (version 7.0-0). Initially, between-study heterogeneity was determined according to Cochran’s Q test statistics. If substantial heterogeneity was identified (*P* < 0.10), the DerSimonian-Laird method fitting the random effects model would be utilized in the pooled effects estimation [34]. Otherwise, without significant heterogeneity (*P* >= 0.10), the Mantel-Haenszel method fitting the fixed effect model would be used [35]. To evaluate the association between GDM risk and the GCKR rs780094 and rs1260326 polymorphisms, the odds ratio (OR) and 95% confidence interval (95% CI) were estimated under the following genetic models: allele model (a *vs.* A), homozygote model (aa *vs.* AA), heterozygote model (Aa *vs.* AA), dominant model (Aa+aa *vs.* AA), and recessive model (aa *vs.* Aa+AA). Forest plots were drawn to display the results of individual datasets and syntheses. Galbraith radial plot was constructed to identify potential outliers as the source of heterogeneity graphically [36, 37]. Publication bias was estimated by the funnel plot asymmetry assessment using Begg’s rank test [38] and Egger’s regression test [39]. *P* < 0.05 was considered a statistically significant asymmetry. The “trim-and-fill” method would be applied to adjust the pooled estimates for the publication bias [40]. “Leave-one-out” sensitivity analysis (remove one dataset from the meta-analysis and re-estimate the pooled effect) was performed to assess the reliance of a meta-analysis on a particular dataset.

All R code and raw data were deposited onto the repository on Gitee forge (https://gitee.com/zhang-yuke128/meta-analysis_gckr_GDM).

## 3 RESULTS

### 3.1 Characteristics of Eligible Studies

**Fig. (1):**
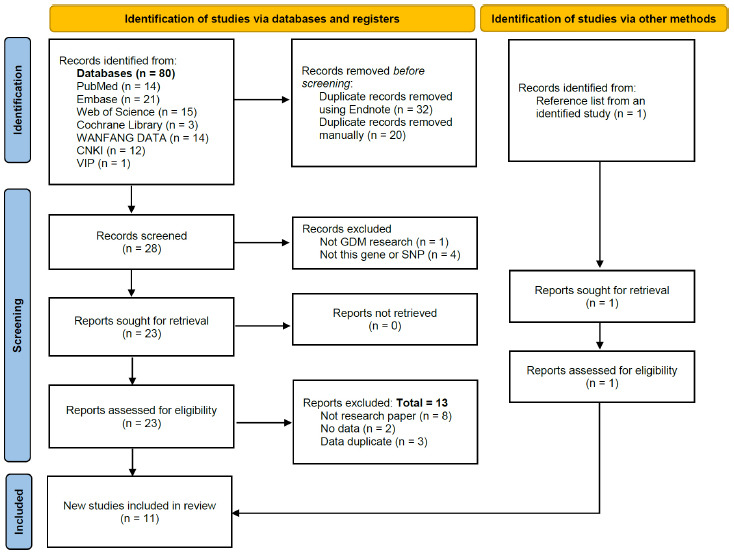
Workflow shows the flow of information through the successive phases of literature selection.

Initially, 80 records were retrieved from all databases. Separately, there were 53 pieces of literature in English (Web of Science: n= 15, PubMed: n= 14, Cochrane Library: n= 3, Embase: n= 21) and 27 Chinese publications (CNKI: n= 12, WANFANG: n= 14, VIP: n= 1). In addition, one Japanese study [24] was identified from the reference list of a previous study [31]. After software-aided automatic and manual duplicate removal, 29 unique studies were kept for further screening. Two studies were excluded because they did not present genotype data [41, 42], and three were eliminated due to data duplication [43-45]. According to the inclusion and exclusion criteria, 11 articles were qualified as finalists for the data extraction phase of this systematic review (nine for rs780094 and four for rs1260326). Fig. **1** graphically summarizes the literature selection process in a PRISMA workflow. The characteristics of the studies are presented in Table **1** and Table **S2**. Ultimately, a total of 13 eligible datasets for rs780094 were included to be reviewed and considered for further statistical analyses [20, 22-29]. The sample sizes range from 134 to 1967, with 9373 participants (3443 GDM cases and 5930 controls). As for rs1260326, five relevant datasets were subjected to the subsequent pooled estimation [21, 27, 28, 30]. The sample sizes fall between 228 and 1663, for a grand total of 4173 subjects (1495 cases and 2678 controls). There was no statistically significant violation of HWE in the control groups, indicating no substantial deviation from HWE in this meta-analysis. In addition, all datasets scored at least five stars in the NOS quality assessment, suggesting medium or good overall methodological quality.

### 3.2 Overall and Subgroup Analysis

**Fig. (2):**
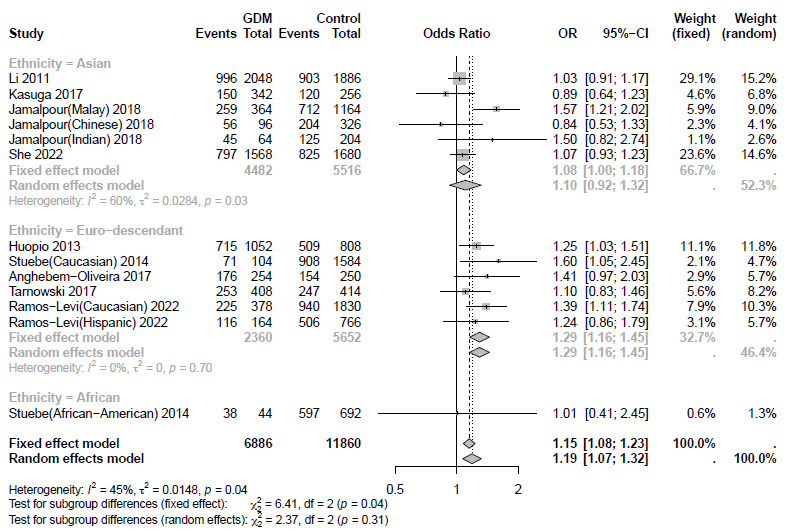
Forest plot for the *GCKR* rs780094 polymorphism and gestational diabetes mellitus risk. The odds ratio (OR) and 95% confidence interval (95% CI) were calculated under the allele model.

For the rs780094 polymorphism, significant between-study heterogeneity was observed in the allele and recessive models. Hence, a random-effects model was fit in these two genetic models to estimate pooled OR and 95% CI, and a fixed-effect model was used in the homozygote, heterozygote, and dominant models. As for the overall population (Table **2**), statistical significance was evident in the allele model (OR: 1.19, 95% CI: 1.07~1.32, *P*< 0.01), homozygote model (OR: 1.27, 95% CI: 1.10~1.47, *P*< 0.01), dominant model (OR: 1.16, 95% CI: 1.03~1.31, *P*= 0.02), and recessive model (OR: 1.31, 95% CI: 1.09~1.57, *P*< 0.01), but not in the heterozygote model (OR: 1.09, 95% CI: 0.96~1.25, *P*= 0.19). After excluding the studies with small sample sizes (less than 300 participants), the statistical significance remained consistent with the overall pooled estimation. In the subgroup analysis of ethnicity (Fig. **2**), significance was only found in the allele model (OR: 1.29, 95% CI: 1.16~1.45, *P*< 0.01), homozygote model (OR: 1.59, 95% CI: 1.19~2.12, *P*< 0.01), dominant model (OR: 1.33, 95% CI: 1.03~1.74, *P*= 0.03), and recessive model (OR: 1.49, 95% CI: 1.23~1.80, *P*< 0.01) of the Euro-descendant subgroup.

**Fig. (3):**
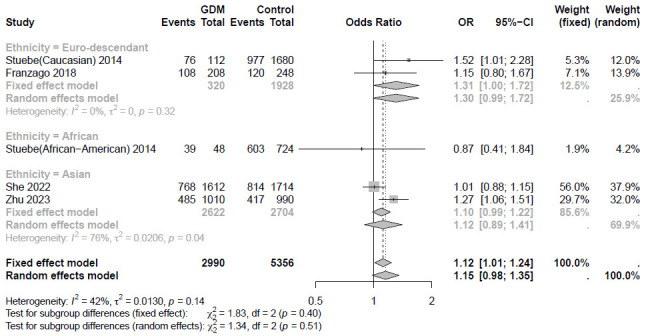
Forest plot for the *GCKR* rs1260326 polymorphism and gestational diabetes mellitus risk. The odds ratio (OR) and 95% confidence interval (95% CI) were calculated under the allele model.

For the rs1260326 polymorphism, no statistical significance was observed in the between-study heterogeneity test. Therefore, the fixed effect model was used for the pooled estimate. In the overall meta-analysis (Table **3**), significant association between this polymorphism and GDM risk was observed in the allele model (OR: 1.12, 95% CI: 1.01~1.24, *P*= 0.03) and homozygote model (OR: 1.26, 95% CI: 1.03~1.54, *P*= 0.02), but not in the heterozygote model (OR: 1.10, 95% CI: 0.93~1.30, *P*= 0.26), dominant model (OR: 1.15, 95% CI: 0.98~1.35, *P*= 0.09), and recessive model (OR: 1.18, 95% CI: 1.00~1.39, *P*= 0.06). In the subgroup analysis of ethnicity (Fig. **3**), marginal significance was achieved in the allele model (OR: 1.31, 95% CI: 1.00~1.72, *P*= 0.05) and homozygote model (OR: 1.84, 95% CI: 1.01~3.34, *P*= 0.05) of the Euro-descendant subgroup. No significance was identified in any subgroup when stratified according to sample size.

### 3.3 Heterogeneity Analysis

**Fig. (4):**
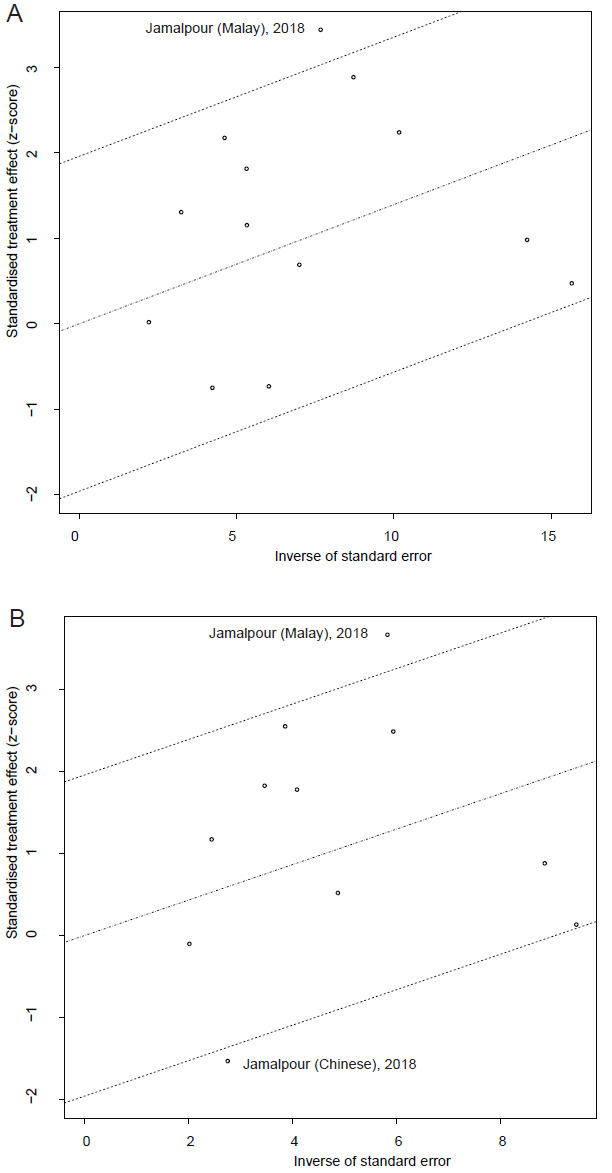
Galbraith plot analysis for the source of between-study heterogeneity in the meta-analysis for the rs780094 polymorphism. (**A**) Outliers were marked with the author’s name in the allele model and (**B**) recessive model .

Galbraith plot assay was performed to find the source of between-study heterogeneity identified in the allele and recessive models of the rs780094 polymorphism. One study in the allele model (Fig. **4A**) and two in the recessive model (Fig. **4B**) were detected as outliers. Once these outlying datasets were excluded, the between-study heterogeneity was significantly eliminated (allele model: *P* = 0.15; recessive model: *P* = 0.16), and the adjusted pooled estimate remained significant (allele model: OR: 1.13, 95% CI: 1.05~1.20, *P*< 0.01; recessive model: OR: 1.20, 95% CI: 1.07~1.35, *P*< 0.01).

### 3.4 Sensitivity Analysis and Publication Bias

“Leave-one-out” sensitivity analysis was performed by iteratively removing one dataset at a time to determine whether any single study could drive the significance of the pooled estimates. For the rs780094 polymorphism (Figs. **S1A**-**E**), the statistical significance of the pooled estimates was maintained in all genetic models except the dominant model (Fig. **S1D**), which missed the statistical significance when the Caucasian cohort from Ramos-Levi’s study [26] was omitted. In addition, the significance became the boundary significance when the Malay cohort from Jamalpour’s study [23] was excluded. For the rs1260326 polymorphism (Figs. **S2A**-**E**), the forest plot illustrated that several datasets could drive the statistical significance of the overall pooled effect estimate in the allele model (Fig. **S2A**) [28, 30], homozygote model (Fig. **S2B**) [28, 30], dominant model (Fig. **S2D**) [27], and recessive model (Fig. **S2E**) [21, 28, 30].

A publication bias assessment was conducted to confirm the reliability of this meta-analysis. A low-level asymmetry was evident from the visual examination of Begg’s funnel plots for the rs780094 (Figs. **S3A**-**E**) and rs1260326 (Figs. **S4A**-**E**) polymorphisms. For the rs780094 polymorphism, neither the Egger’s regression test (allele model: *P*= 0.290, homozygote model: *P*= 0.224, heterozygote model: *P*= 0.761, dominant model: *P*= 0.253, recessive model: *P*= 0.433) nor Begg’s rank test (allele model: *P*= 0.669, homozygote model: *P*= 0.876, heterozygote model: *P*= 0.876, dominant model: *P*= 0.533, recessive model: *P*= 0.876) achieved statistical significance. For the rs1260326 polymorphism, all *P*-values derived from Egger’s regression test (allele model: *P*= 0.629, homozygote model: *P*= 0.709, heterozygote model: *P*= 0.679, dominant model: *P*= 0.663, recessive model: *P*= 0.695) and Begg’s rank test (allele model: *P*= 1.000, homozygote model: *P*= 1.000, heterozygote model: *P*= 0.462, dominant model: *P*= 0.807, recessive model: *P*= 0.807) were greater than 0.05. Both the symmetry of Begg’s funnel plots and the non-significant result of asymmetry inspection suggested a low possibility of publication bias for the GDM studies of both the rs780094 and rs1260326 polymorphisms.

## 4 DISCUSSION

In this meta-analysis, we integrated 11 studies to clarify whether the GCKR rs780094 and rs1260326 polymorphic variants were differentially represented among GDM patients and the control subjects, which were consistent with previous clinical observations for the impact of these two polymorphisms on deviant glucose homeostasis, hypertriglyceridemia, and insulin resistance [9-11]. For the rs780094 polymorphism, a statistically significant association was observed in the allele, homozygote, dominant, and recessive models. Further subgroup analysis by ethnicity showed that the significance was only present in the Euro-descendant subgroup, not the others. This indicates the possible association between this variant and the increased GDM risk in populations with European ancestry (*e.g.,* Caucasian and Hispanic). However, genetic factors within certain ethnicities could not fully explain the susceptibility trend. Further well-designed prospective longitudinal studies should also investigate its interaction with environmental exposure and lifestyle effects. For the rs1260326 polymorphism, statistical significance was identified in the allele and homozygote models. However, when stratified according to ethnicity, no subgroup showed significance. Considering the small sample size of the overall population and individual subgroups, more studies with large-scale sample sizes should be performed to further validate the causal association.

A qualified meta-analysis should not only statistically summarize the overall effect but also illustrate how trustworthy this pooled estimation is [46]. Assessing the magnitude of between-study heterogeneity in a systematic review and meta-analysis is essential for determining the appropriateness of pooled results [46, 47]. Considerable heterogeneity was evident in the allele and recessive models for the rs780094 polymorphism. Studies with small sample sizes tend to be more heterogeneous than larger ones [48]. However, removing studies with less than 300 participants did not relieve the heterogeneity. Then, a Galbraith plot assay identified the outliers contributing to the significant between-study heterogeneity. The exclusion of these outlying studies substantially mitigated the heterogeneity, and the adjusted effect size remained statistically significant, suggesting the reliability of the meta-analytic results.

The merits of this systematic review and meta-analysis are apparent. No significant deviation from the HWE expectations in the control groups among the included datasets was identified, reflecting a low hazard of selection bias, population stratification, and genotyping errors [49]. Meanwhile, the NOS quality assessment found no studies with low scores, indicating at least a medium level of methodological quality. These two advantages ensured the incorporated datasets' quality and enhanced the credibility of the pooled estimates. Furthermore, no significant publication bias was evident, indicating the low likelihood of the overestimated effect sizes and the dissemination of false-positive outcomes [50]. This ruled out the possibility of the distortion of the existence and magnitude of the pathogenic effect led by the two GCKR genetic polymorphisms. Moreover, compared with previous studies [31, 32], the computerized systematic query in seven literature databases in this meta-analysis substantially expanded the search scope. It lowered the probability of omission of relevant datasets. Last but not least, the detailed flow of this meta-analysis followed close to the PRISMA recommendations [33], which normalized the procedures of conception and design, team organization, literature search and selection, statistical analysis, results interpretation, and manuscript drafting.

The limitations of this meta-analysis should be acknowledged. First, the language limitation of the authors restricted the inclusion of relevant studies not published in Chinese and English. Second, the participants involved in this meta-analysis were predominantly of Caucasian and Asian ethnicities, which substantially limited the generality of the conclusions proposed in this meta-analysis. Third, significant between-study heterogeneity is an undeniable fact in this meta-analysis, which could limit the extent to which generalizable inferences can be drawn [51]. Fourth, the findings of the sensitivity analysis for both polymorphisms show that several single studies drove the statistical significance of the pooled results in some genetic models. Fifth, this meta-analysis is not registered, and the protocol is not prepared. Finally, the number of included studies and sample size for the rs1260326 polymorphism are still relatively small, which inevitably undermines the statistical power of the pooled estimate.

## CONCLUSION

Considering all, this meta-analysis intended to disclose the possible causal association of the GCKR rs780094 and rs1260326 polymorphisms with increased risk of GDM by pooling the available data. However, further large-scale validation is necessary to provide consistent and conclusive evidence for their implication in GDM pathogenesis.

## Figures and Tables

**Table 1:** Characteristics of included studies.

**First Author**	**Year**	**Country** **(Ethnicity)**	**Genotyping Method**	**Diagnostic Method**	**Case** **(RAF)**	**Control** **(RAF)**	**HWE**	**NOS**
**rs780094**								
Li	2011	China (Asian)	TaqMan	GCT+OGTT	0.49	0.48	Meet	6
Huopio	2013	Finland (Caucasian)	NA	OGTT	0.68	0.63	Meet	6
Stuebe	2014	US (Caucasian)	IPLEXMassARRAY PCR	GLT+OGTT	0.68	0.57	Meet	6
Stuebe	2014	US (African-American)	IPLEXMassARRAY PCR	GLT+OGTT	0.86	0.86	Meet	5
Kasuga	2017	Japan (Asian)	Sequenom MassARRAY	IADPSG	0.44	0.47	Meet	7
Anghebem-Oliveira	2017	Brazil (Euro-Brazilian)	TaqMan	ABDA	0.69	0.62	Meet	6
Tarnowski	2017	Poland (Caucasian)	TaqMan	IADPSG	0.62	0.60	Meet	6
Jamalpour	2018	Malaysia (Malayan)	IPLEXMassARRAY PCR	modified OGTT	0.71	0.61	Meet	6
Jamalpour	2018	Malaysia (Malaysian Chinese)	IPLEXMassARRAY PCR	modified OGTT	0.58	0.63	Meet	5
Jamalpour	2018	Malaysia (Malaysian Indian)	IPLEXMassARRAY PCR	modified OGTT	0.70	0.61	Meet	5
She	2022	China (Asian)	Sequenom MassARRAY	IADPSG	0.51	0.49	Meet	6
Ramos-Levi	2022	Spain (Caucasian)	IPLEXMassARRAY PCR	IADPSG	0.60	0.51	Meet	6
Ramos-Levi	2022	Spain (Hispanic)	IPLEXMassARRAY PCR	IADPSG	0.71	0.66	Meet	6
**rs1260326**								
Stuebe	2014	US (Caucasian)	IPLEXMassARRAY PCR	GLT+OGTT	0.68	0.58	Meet	6
Stuebe	2014	US (African-American)	IPLEXMassARRAY PCR	GLT+OGTT	0.81	0.83	Meet	5
Franzago	2018	Italy (Caucasian)	Sequencing	IADPSG	0.52	0.48	Meet	7
She	2022	China (Asian)	Sequenom MassARRAY	IADPSG	0.48	0.47	Meet	6
Zhu	2023	China (Asian)	Sequencing	OGTT	0.48	0.42	Meet	6

**Note:** * RAF= risk allele frequency; HWE= Hardy-Weinberg equilibrium; NOS= Newcastle-Ottawa Quality Assessment Scale; TaqMan=TaqMan SNP Genotyping Assays; PCR=polymerase chain reaction; NA=not available; GCT=glucose challenge test; OGTT=oral glucose tolerance test; GLT=glucose loading test; IADPSG=the International Association of Diabetes and Pregnancy Study Groups; ABDA=2015 criteria of the American and Brazilian Diabetes Associations.

**Table 2:** Overall and stratified meta-analysis results for the association between the GCKR rs780094 polymorphism and gestational diabetes mellitus risk.

**Genetic Model**	**No. of** **Datasets**	**No. of Cases**	**No. of** **Controls**	***P*h**	**Effect Model**	**OR (95% CI)**	***P-*value**
**Total**							
Allele	13	6886	11860	0.04	Random	1.19 (1.07, 1.32)	<0.01
Homozygote	11	1470	2889	0.28	Fixed	1.27 (1.10, 1.47)	<0.01
Heterozygote	11	1868	3592	0.76	Fixed	1.09 (0.96, 1.25)	0.19
Dominant	11	2746	5398	0.67	Fixed	1.16 (1.03, 1.31)	0.02
Recessive	11	2746	5398	0.01	Random	1.31 (1.09, 1.57)	<0.01
**Asian**							
Allele	6	4482	5516	0.03	Random	1.10 (0.92, 1.32)	0.23
Homozygote	6	1085	1369	0.23	Fixed	1.18 (1.00, 1.39)	0.06
Heterozygote	6	1486	1876	0.87	Fixed	1.08 (0.93, 1.26)	0.30
Dominant	6	2070	2630	0.67	Fixed	1.11 (0.97, 1.28)	0.13
Recessive	6	2070	2630	<0.01	Random	1.17 (0.83, 1.64)	0.29
**African**							
Allele	1	44	692	NA	NA	1.01 (0.41, 2.45)	0.99
Homozygote	1	16	259	NA	NA	0.58 (0.03, 11.26)	0.72
Heterozygote	1	6	91	NA	NA	0.67 (0.03, 13.82)	0.79
Dominant	1	22	346	NA	NA	0.59 (0.03, 11.33)	0.73
Recessive	1	22	346	NA	NA	0.95 (0.36, 2.51)	0.92
**Euro-descendant**							
Allele	6	2360	5652	0.70	Fixed	1.29 (1.16, 1.45)	<0.01
Homozygote	4	369	1261	0.51	Fixed	1.59 (1.19, 2.12)	<0.01
Heterozygote	4	376	1625	0.27	Fixed	1.14 (0.86, 1.50)	0.38
Dominant	4	654	2422	0.42	Fixed	1.33 (1.03, 1.74)	0.03
Recessive	4	654	2422	0.51	Fixed	1.49 (1.23, 1.80)	<0.01
**>= 300**							
Allele	9	6130	10824	0.05	Random	1.21 (1.08, 1.36)	<0.01
Homozygote	8	1354	2690	0.13	Fixed	1.27 (1.09, 1.47)	<0.01
Heterozygote	8	1754	3348	0.78	Fixed	1.09 (0.96, 1.25)	0.19
Dominant	8	2539	5008	0.46	Fixed	1.16 (1.02, 1.31)	0.02
Recessive	8	2539	5008	0.04	Random	1.30 (1.08, 1.57)	<0.01
**< 300**							
Allele	4	756	1036	0.13	Fixed	1.07 (0.88, 1.31)	0.50
Homozygote	3	116	199	0.64	Fixed	1.38 (0.76, 2.50)	0.29
Heterozygote	3	114	244	0.28	Fixed	1.07 (0.60, 1.88)	0.83
Dominant	3	207	390	0.66	Fixed	1.21 (0.70, 2.10)	0.49
Recessive	3	207	390	0.02	Random	1.24 (0.59, 2.61)	0.58

**Note:** * *P*h = *P*-value of heterogeneity.

**Table 3:** Overall and stratified meta-analysis results for the association between the GCKR rs1260326 polymorphism and gestational diabetes mellitus risk.

**Genetic Model**	**No. of** **Datasets**	**No. of Cases**	**No. of** **Controls**	***P*h**	**Effect Model**	**OR (95% CI)**	***P-*value**
**Total**							
Allele	5	2990	5356	0.14	Fixed	1.12 (1.01, 1.24)	0.03
Homozygote	5	759	1439	0.10	Fixed	1.26 (1.03, 1.54)	0.02
Heterozygote	5	1125	1832	0.64	Fixed	1.10 (0.93, 1.30)	0.26
Dominant	5	1495	2678	0.34	Fixed	1.15 (0.98, 1.35)	0.09
Recessive	5	1495	2678	0.21	Fixed	1.18 (1.00, 1.39)	0.06
**Asian**							
Allele	2	2622	2704	0.04	Random	1.12 (0.89, 1.41)	0.32
Homozygote	2	666	683	0.03	Random	1.27 (0.79, 2.04)	0.33
Heterozygote	2	1007	1071	0.60	Fixed	1.07 (0.90, 1.28)	0.46
Dominant	2	1311	1352	0.23	Fixed	1.11 (0.94, 1.32)	0.21
Recessive	2	1311	1352	0.03	Random	1.21 (0.80, 1.83)	0.37
**African**							
Allele	1	48	724	NA	NA	0.87 (0.41, 1.84)	0.72
Homozygote	1	17	255	NA	NA	0.45 (0.05, 3.90)	0.47
Heterozygote	1	8	114	NA	NA	0.46 (0.05, 4.26)	0.49
Dominant	1	24	362	NA	NA	0.45 (0.05, 3.84)	0.47
Recessive	1	24	362	NA	NA	0.92 (0.83, 2.21)	0.85
**Euro-descendant**							
Allele	2	320	1928	0.32	Fixed	1.31 (1.00, 1.72)	0.05
Homozygote	2	76	501	0.31	Fixed	1.84 (1.01, 3.34)	0.05
Heterozygote	2	110	647	0.40	Fixed	1.46 (0.85, 2.50)	0.17
Dominant	2	160	964	0.30	Fixed	1.58 (0.94, 2.63)	0.08
Recessive	2	160	964	0.56	Fixed	1.37 (0.91, 2.06)	0.14
**>= 300**							
Allele	4	2782	5108	0.08	Random	1.16 (0.95, 1.41)	0.14
Homozygote	4	713	1383	0.05	Random	1.36 (0.87, 2.11)	0.18
Heterozygote	4	1046	1734	0.49	Fixed	1.09 (0.92, 1.30)	0.32
Dominant	4	1391	2554	0.22	Fixed	1.14 (0.97, 1.35)	0.11
Recessive	4	1391	2554	0.12	Fixed	1.17 (0.99, 1.39)	0.07
**< 300**							
Allele	1	208	248	NA	NA	1.15 (0.80, 1.67)	0.45
Homozygote	1	46	56	NA	NA	1.37 (0.63, 3.00)	0.43
Heterozygote	1	79	98	NA	NA	1.22 (0.63, 2.35)	0.56
Dominant	1	104	124	NA	NA	1.26 (0.67, 2.37)	0.47
Recessive	1	104	124	NA	NA	1.19 (0.64, 2.23)	0.58

**Note:** * *P* h = *P*-value of heterogeneity.

## LIST OF ABBREVIATIONS

CI: Confidential Interval
CNKI: Chinese National Knowledge Infrastructure
GCK: Glucokinase
GCKR: Glucokinase Regulator Gene
GDM: Gestational Diabetes Mellitus
GKRP: Glucokinase Regulatory Protein
HWE: Hardy-Weinberg Equilibrium
NOS: Newcastle-Ottawa Scale
OR: Odds Ratio
PRISMA: Preferred Reporting Items for Systematic Reviews and Meta-Analyses
SNP: Single Nucleotide Polymorphism
T2DM: Type 2 Diabetes Mellitus
VIP: China Science and Technology Journal Database

## STANDARDS OF REPORTING

PRISMA guidelines and methodology were followed while conducting the study.

## SUPPLEMENTARY MATERIAL

PRISMA checklist is available as supplementary material on the publisher’s website along with the published article. Supplementary material is available on the publisher’s website along with the published article.
