# Supplementary material for: Contribution of Type 2 Diabetes Susceptible Gene GCKR Polymorphisms Rs780094 and Rs1260326 to Gestational Diabetes Mellitus: A Meta-Analysis
Source: Endocr Metab Immune Disord Drug Targets. 2025 Jan 9;26:E18715303313654. doi: 10.2174/0118715303313654241101042033 (PMC13284661; doi:10.2174/0118715303313654241101042033)
Supplement: Supplementary file 1 [file EMIDDT-26-E18715303313654_SD1.pdf]

SUPPLEMENTARY MATERIAL

Contribution of Type 2 Diabetes Susceptible Gene GCKR Polymorphisms Rs780094 and Rs1260326 to Gestational Diabetes Mellitus: A Meta-Analysis

Yuke Zhang[1, #], Kuangyi Wang[2, #], Chenxi Ji[1, #], Yansi Lin[1, #], Zitong Liu[1, #], Jing Chen[1, #], Feifei Zheng[1], Xiaoqin Yang[1,*], Yi Sun[3,*]

[1]Center for Systems Biology, Department of Bioinformatics, School of Biology and Basic Medical Sciences, Suzhou Medical College of Soochow University, Suzhou 215123, China; [2]Department of Cardiology, the Fourth Affiliated Hospital of Soochow University and Medical Center of Soochow University Suzhou 215123, China; [3]Department of Genetics, School of Biology and Basic Medical Sciences, Suzhou Medical College of Soochow University, Suzhou 215123, China.

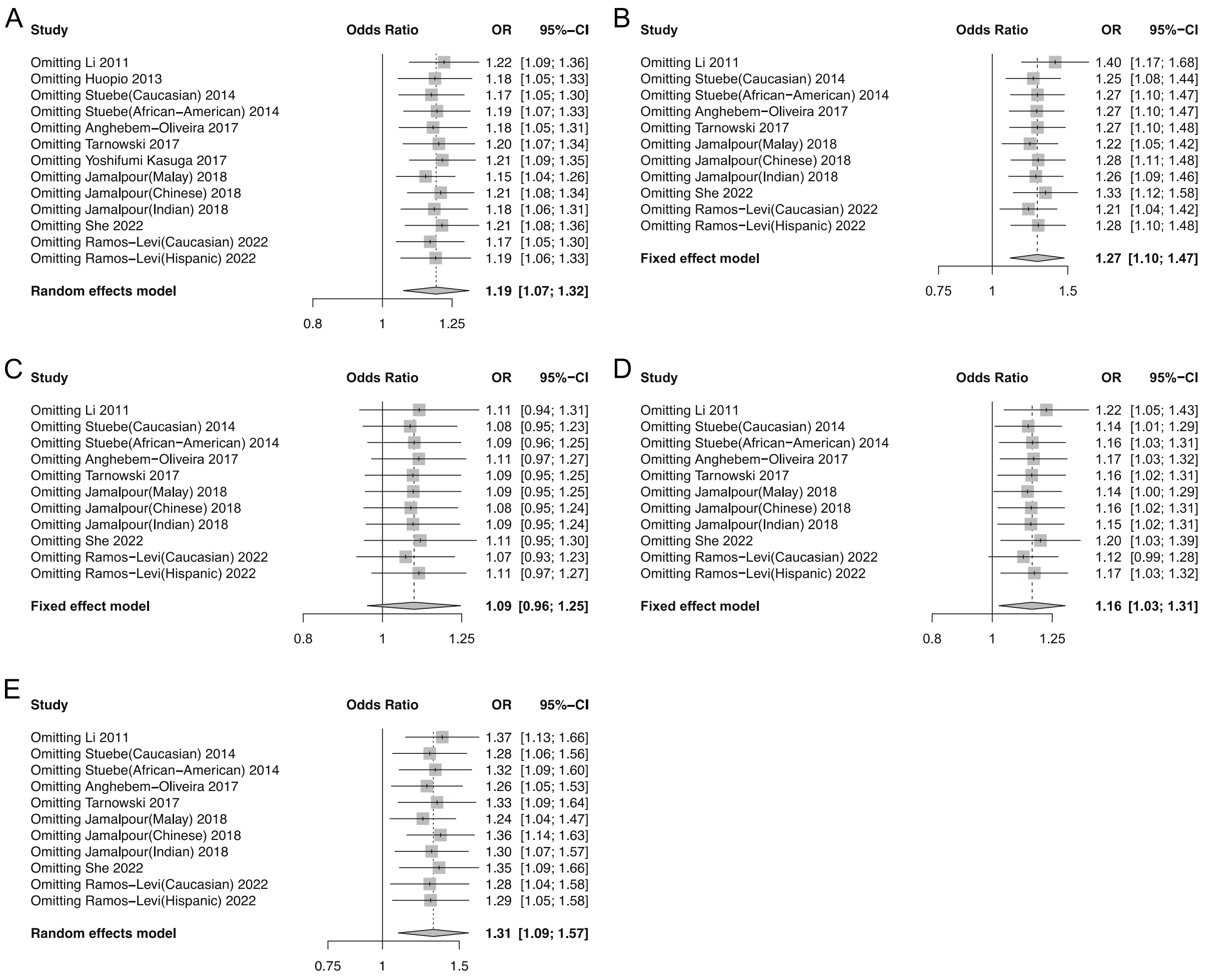

Fig. (S1). Sensitivity analysis for the *GCKR* rs780094 polymorphism. (**A**) Allele model. (**B**) Homozygote model. (**C**) Heterozygote model. (**D**) Dominant model. (**E**) Recessive model.

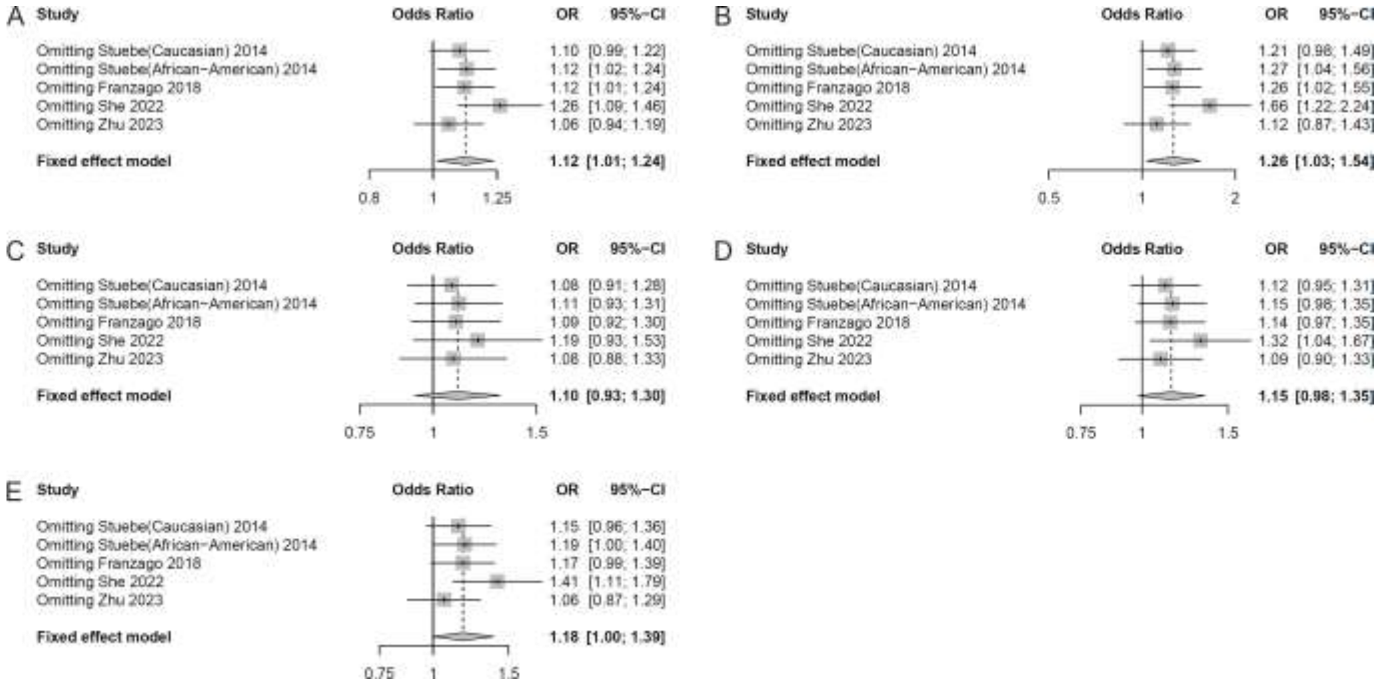

Fig. (S2). Sensitivity analysis for the *GCKR* rs1260326 polymorphism. (**A**) Allele model. (**B**) Homozygote model. (**C**) Heterozygote model. (**D**) Dominant model. (**E**) Recessive model.

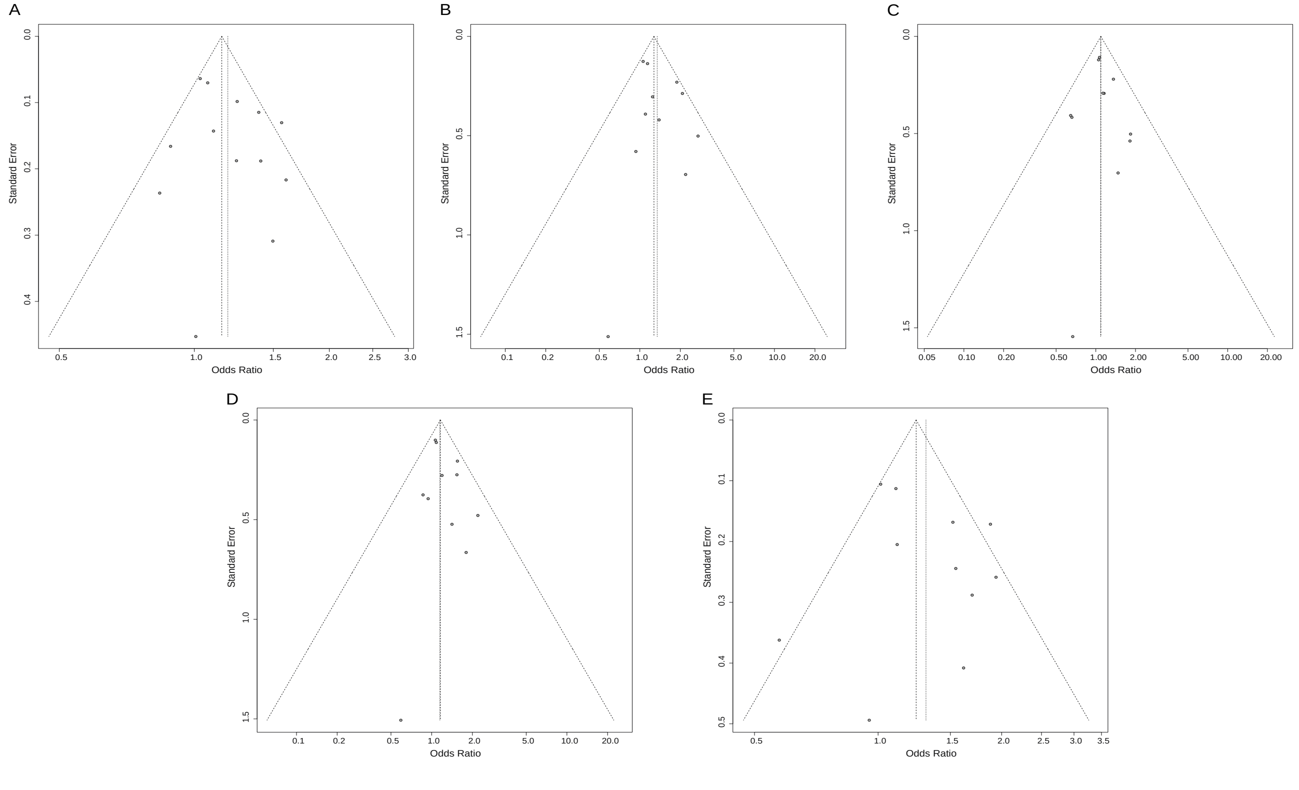

Fig. (S3). Publication bias for the *GCKR* rs780094 polymorphism. (**A**) Allele model. (**B**) Homozygote model. (**C**) Heterozygote model. (**D**) Dominant model. (**E**) Recessive model.

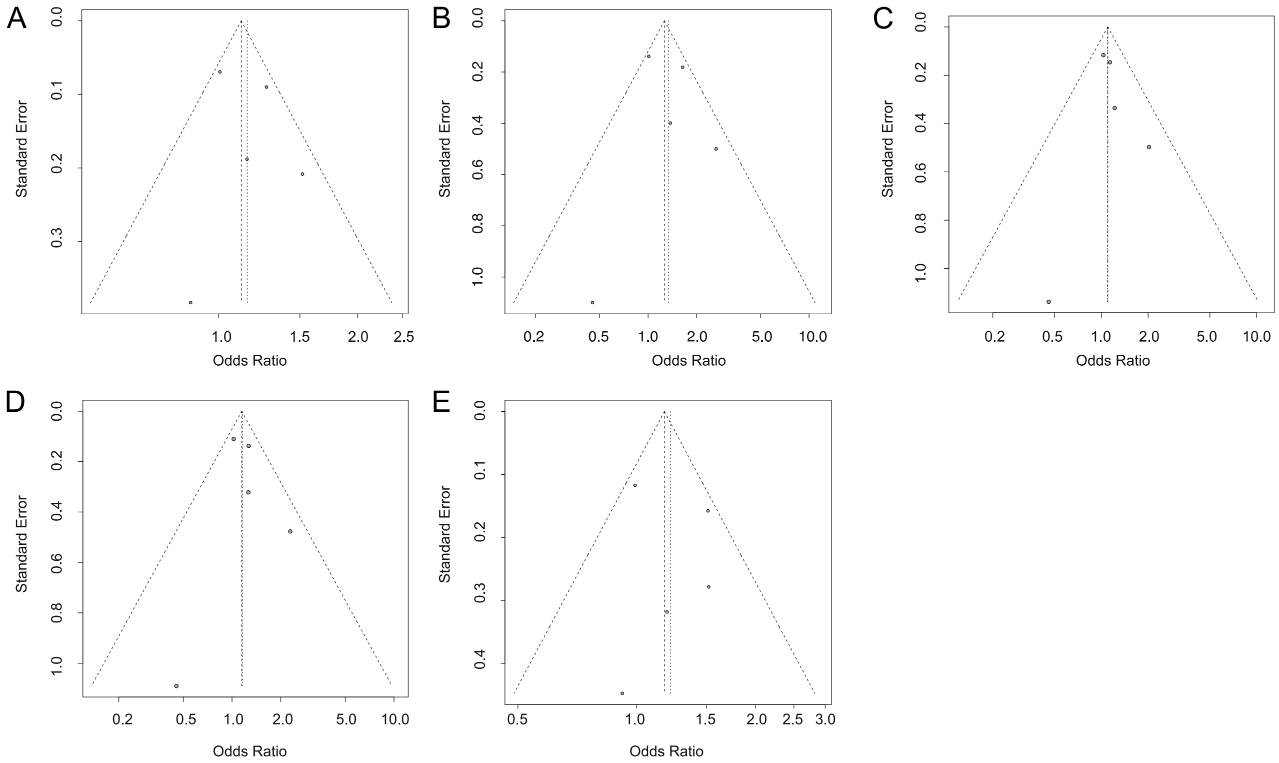

Fig. (S4). Publication bias for the *GCKR* rs1260326 polymorphism. (**A**) Allele model. (**B**) Homozygote model. (**C**) Heterozygote model. (**D**) Dominant model. (**E**) Recessive model.

Table S1. Checklist of Preferred Reporting Items for Systematic Reviews and Meta-Analyses (PRISMA).

PRISMA 2020 Checklist

Section and Topic	Item #	Checklist item	Location where item is reported
**TITLE**			
Title	1	Identify the report as a systematic review.	1
**ABSTRACT**			
Abstract	2	See the PRISMA 2020 for Abstracts checklist.	1
**INTRODUCTION**			
Rationale	3	Describe the rationale for the review in the context of existing knowledge.	1,2
Objectives	4	Provide an explicit statement of the objective(s) or question(s) the review addresses.	2
**METHODS**			
Eligibility criteria	5	Specify the inclusion and exclusion criteria for the review and how studies were grouped for the syntheses.	2
Information sources	6	Specify all databases, registers, websites, organisations, reference lists and other sources searched or consulted to identify studies. Specify the date when each source was last searched or consulted.	2
Search strategy	7	Present the full search strategies for all databases, registers and websites, including any filters and limits used.	2
Selection process	8	Specify the methods used to decide whether a study met the inclusion criteria of the review, including how many reviewers screened each record and each report retrieved, whether they worked independently, and if applicable, details of automation tools used in the process.	2
Data collection process	9	Specify the methods used to collect data from reports, including how many reviewers collected data from each report, whether they worked independently, any processes for obtaining or confirming data from study investigators, and if applicable, details of automation tools used in the process.	2
Data items	10a	List and define all outcomes for which data were sought. Specify whether all results that were compatible with each outcome domain in each study were sought (e.g. for all measures, time points, analyses), and if not, the methods used to decide which results to collect.	2
	10b	List and define all other variables for which data were sought (e.g. participant and intervention characteristics, funding sources). Describe any assumptions made about any missing or unclear information.	2
Study risk of bias assessment	11	Specify the methods used to assess risk of bias in the included studies, including details of the tool(s) used, how many reviewers assessed each study and whether they worked independently, and if applicable, details of automation tools used in the process.	2
Effect measures	12	Specify for each outcome the effect measure(s) (e.g. risk ratio, mean difference) used in the synthesis or presentation of results.	3
Synthesis methods	13a	Describe the processes used to decide which studies were eligible for each synthesis (e.g. tabulating the study intervention characteristics and comparing against the planned groups for each synthesis (item #5)).	2,3
	13b	Describe any methods required to prepare the data for presentation or synthesis, such as handling of missing summary statistics, or data conversions.	2,3
	13c	Describe any methods used to tabulate or visually display results of individual studies and syntheses.	3
	13d	Describe any methods used to synthesize results and provide a rationale for the choice(s). If meta-analysis was performed, describe the model(s), method(s) to identify the presence and extent of statistical heterogeneity, and software package(s) used.	3
	13e	Describe any methods used to explore possible causes of heterogeneity among study	3

		results (e.g. subgroup analysis, meta-regression).	
	13f	Describe any sensitivity analyses conducted to assess robustness of the synthesized results.	3
Reporting bias assessment	14	Describe any methods used to assess risk of bias due to missing results in a synthesis (arising from reporting biases).	3
Certainty assessment	15	Describe any methods used to assess certainty (or confidence) in the body of evidence for an outcome.	NA
**RESULTS**			
Study selection	16a	Describe the results of the search and selection process, from the number of records identified in the search to the number of studies included in the review, ideally using a flow diagram.	3
	16b	Cite studies that might appear to meet the inclusion criteria, but which were excluded, and explain why they were excluded.	3
Study characteristics	17	Cite each included study and present its characteristics.	3,4
Risk of bias in studies	18	Present assessments of risk of bias for each included study.	4
Results of individual studies	19	For all outcomes, present, for each study: (a) summary statistics for each group (where appropriate) and (b) an effect estimate and its precision (e.g. confidence/credible interval), ideally using structured tables or plots.	5-8
Results of syntheses	20a	For each synthesis, briefly summarise the characteristics and risk of bias among contributing studies.	3,4
	20b	Present results of all statistical syntheses conducted. If meta-analysis was done, present for each the summary estimate and its precision (e.g. confidence/credible interval) and measures of statistical heterogeneity. If comparing groups, describe the direction of the effect.	4-6
	20c	Present results of all investigations of possible causes of heterogeneity among study results.	6-8
	20d	Present results of all sensitivity analyses conducted to assess the robustness of the synthesized results.	7
Reporting biases	21	Present assessments of risk of bias due to missing results (arising from reporting biases) for each synthesis assessed.	7
Certainty of evidence	22	Present assessments of certainty (or confidence) in the body of evidence for each outcome assessed.	NA
**DISCUSSION**			
Discussion	23a	Provide a general interpretation of the results in the context of other evidence.	8,9
	23b	Discuss any limitations of the evidence included in the review.	9
	23c	Discuss any limitations of the review processes used.	9
	23d	Discuss implications of the results for practice, policy, and future research.	9
**OTHER INFORMATION**			
Registration and protocol	24a	Provide registration information for the review, including register name and registration number, or state that the review was not registered.	9
	24b	Indicate where the review protocol can be accessed, or state that a protocol was not prepared.	9
	24c	Describe and explain any amendments to information provided at registration or in the protocol.	NA
Support	25	Describe sources of financial or non-financial support for the review, and the role of the funders or sponsors in the review.	10
Competing interests	26	Declare any competing interests of review authors.	10
Availability of data, code and other materials	27	Report which of the following are publicly available and where they can be found: template data collection forms; data extracted from included studies; data used for all analyses; analytic code; any other materials used in the review.	3

From: Page MJ, McKenzie JE, Bossuyt PM, Boutron I, Hoffmann TC, Mulrow CD, et al. The PRISMA 2020 statement: an updated guideline for reporting systematic reviews. BMJ 2021;372:n71. doi: 10.1136/bmj.n71
For more information, visit: http://www.prisma-statement.org/

Table S2. Genotype data of the *GCKR* rs780094 and rs1260326 polymorphisms included in this meta-analysis.

Reference	Year	Country (Ethnicity)	Case					Control				
			TT	CT	CC	T	C	TT	CT	CC	T	C
**rs780094**												
Li	2011	China(Asian)	275	502	247	1052	996	265	453	225	983	903
Huopio	2013	Finland(Caucasian)	NA	NA	NA	337	715	NA	NA	NA	299	509
Stuebe	2014	US(Caucasian)	5	23	24	33	71	150	376	266	676	908
Stuebe	2014	US(African-American)	0	6	16	6	38	4	87	255	95	597
Kasuga	2017	Japan(Asian)	NA	NA	NA	192	150	NA	NA	NA	136	120
Anghebem-Oliveira	2017	Brazil(Euro-Brazilian)	15	48	64	78	176	14	68	43	96	154
Tarnowski	2017	Poland(Caucasian)	28	99	77	155	253	33	101	73	167	247
Jamalpour	2018	Malaysia(Malayan)	18	69	95	105	259	84	284	214	452	712
Jamalpour	2018	Malaysia(Malaysian Chinese)	5	30	13	40	56	23	76	64	122	204
Jamalpour	2018	Malaysia(Malaysian Indian)	3	13	16	19	45	16	47	39	79	125
She	2022	China(Asian)	200	371	213	771	797	227	401	212	855	825
Ramos-Levi	2022	Spain(Caucasian)	33	87	69	153	225	226	438	251	890	940
Ramos-Levi	2022	Spain(Hispanic)	10	28	44	48	116	41	178	164	260	506
**rs1260326**												
Stuebe	2014	US(Caucasian)	5	26	25	36	76	154	395	291	703	977
Stuebe	2014	US(African-American)	1	7	16	9	39	7	107	248	121	603
Franzago	2018	Italy(Caucasian)	21	58	25	100	108	30	68	26	128	120
She	2022	China(Asian)	220	404	182	844	768	238	424	195	900	814
Zhu	2023	China(Asian)	142	241	122	525	485	164	245	86	573	417
